# Magnetic Resonance Susceptibility-Weighted Imaging versus Other Imaging Modalities in Detecting Splenic Siderotic Lesions

**DOI:** 10.1371/journal.pone.0073626

**Published:** 2013-09-09

**Authors:** Chuanming Li, Daiquan Zhou, Jun Zhao, Xin Wang, Wei Mu, Jian Wang

**Affiliations:** Department of Radiology, Southwest Hospital, Third Military Medical University, Chongqing, China; Wayne State University, United States of America

## Abstract

**Background:**

Susceptibility-weighted imaging (SWI) has been proven to be superior to T2*-weighted imaging and also other existing magnetic resonance imaging (MRI) techniques for the detection of iron content and hemorrhage in the brain. The purpose of this study was to compare SWI with T1WI, T2WI and T2*WI in detecting splenic siderotic lesions.

**Methodology/Principal Findings:**

Twenty-two patients with splenic siderotic nodule were imaged with non-contrast MRI T1WI, T2WI, T2*WI and SWI at 3.0 Tesla. Imaging data were independently analyzed by two experienced radiologists. The number of splenic siderotic nodules was counted, and the size (largest diameter) was measured. The conspicuity was calculated as the nodule to background parenchyma intensity ratio. We found that SWI detected a larger average number of splenic siderotic nodules than T1WI, T2WI, or T2*WI (all *P*<0.05). The average size of the nodules detected by SWI was larger than that of those detected by T1WI, T2WI or T2*WI (all *P*<0.05). SWI provided superior contrast and visibility for splenic siderotic nodules compared to any other sequence (all *P*<0.001).

**Conclusions:**

SWI may be a better detection scheme for splenic siderotic nodules than T1WI, T2WI and T2*WI.

## Introduction

Iron is a critical element for human life. Abnormalities of iron metabolism are associated with the formation of siderotic nodules in the spleen, liver and bone marrow, contributing to numerous diseases such as hepatic cirrhosis, thalassemia, hemochromatosis, sickle cell disease, and aplastic anemia [1]–[4].

Because of their clinical importance, many techniques have been developed to detect hepatic and splenic siderotic nodules. Ultrasound lacks the ability to discriminate iron contents. It is notorious for user dependents and can be particularly difficult with obese patients. In comparison, computed tomography (CT) produces high-quality images, especially with the enhancement multi-detector CT and contrast agents. However, CT also has limited sensitivity for the detection of iron and has several disadvantages including radiation exposure, anaphylactic shock and nephrotoxic impairment [5], [6]. T2*-weighted magnetic resonance (MR) imaging is considered to be the most sensitive medical imaging technique for the measurement of iron content. It is based on local field inhomogeneity generated mostly by the paramagnetic effect of hemosiderin particles. Over the last two decades, many investigations have been performed using gradient-echo sequences, with good results overall [7]–[10].

By adding phase information, susceptibility-weighted imaging (SWI) achieves even greater sensitivity for detecting focal iron than T2* contrast imaging. It has been well documented that SWI is superior to T2*WI and to other existing magnetic resonance imaging (MRI) techniques for the detection of iron content and hemorrhage in the brain [11], [12]. The purpose of this study was to evaluate the value of abdominal SWI for the detection of splenic siderotic nodules compared with T1WI, T2WI and T2*WI.

## Materials and Methods

### Ethics Statement

All research procedures were approved by the Institutional Review Board of our institution and were conducted in accordance with the Declaration of Helsinki. Written informed consent was obtained for all patients prior to the study.

### Subjects

From March 2011 to December 2012, twenty-six consecutive patients participated in this study at our hospital. Patient inclusion criteria were as follows: 1) Patient were able to sustain a 20-second breath hold; 2) Patient age was between 18 years and 80 years; 3) Patient had at least one visible splenic siderotic nodule previously detected by MR; and 4) no other lesions in the spleen were found by previously MR. The diagnosis of siderotic nodules was based on hypointense on MRI compared to background parenchyma excluding cross sections of blood vessels in the spleen [13], [14]. Patients were excluded from the study if they were pregnant, had an implanted device, or had any contraindication for MRI (n = 4). A total of 22 patients met the inclusion criteria and were enrolled in the study (10 men and 12 women; mean age, 38.8±16.5 years).

### Magnetic Resonance Imaging

For all subjects, MR imaging of the upper abdomen, without intravenous or oral contrast enhancement, was performed on a 3.0-T whole body system (Magnetom Trio, Siemens Healthcare, Erlangen, Germany) equipped with a standard 12-channel matrix coil. The imaging sequence included transverse T1-weighted 2D gradient echo (GRE) (flip angle 70°, TR/TE 140/2.46 ms), transverse T2-weighted 2D fast spin echo (flip angle 122°, TR/TE = 3700/84 ms, ETL 9), transverse T2*-weighted 2D GRE (flip angle 20°, TR/TE = 150/10 ms) and transverse abdominal 2D SWI (flip angle 20°, TR/TE = 150/10 ms). For all sequences, field of view (FOV) was 380 × 285 mm^2^, the matrix was 384 × 250, and slice thickness was 5 mm with a 1 mm gap. Two to three breath holds of 15- to 20-s duration were used for each sequence. The recently developed multislice 2D GRE sequence with SWI reconstruction (Siemens work-in-progress sequence #608, Siemens Healthcare, Erlangen, Germany) was used to perform abdominal SWI. SWI post-processing was done online using the following procedure: 1) Original images from each channel were passed through a 32 × 32 high pass filter to remove background artifacts; 2) Modulus-weighted magnitude and phase images were combined into a complex image; 3) High pass filter corrected phase images were created from the complex images; and 4) a normalized phase mask was calculated from each corrected phased image and multiplied with the magnitude image to produce the final SWI and phase image [15].

### Image Analysis

Two radiologists, with 10 and 12 years of experience in abdominal imaging, independently analyzed the images. They were blinded to previous clinical MR interpretations, clinical history and pathologic results. Based on a previous study, spots that were hypointense relative to background spleen parenchyma on T1-, T2-, T2*- weighted, and SWI images were considered siderotic nodules after excluding cross sections of blood vessels in the spleen [13], [14]. By reader consensus, the single representative slice that contained the largest number of splenic siderotic nodules was chosen from the SWI dataset. After corresponding slices were chosen from the T1WI, T2WI and T2*WI datasets, all slices were compared. The number of visible splenic siderotic nodules was counted and the size (largest diameter) was measured. The conspicuity of splenic siderotic nodules on MRI was calculated as the ratio of nodule signal intensity to background parenchyma signal intensity. Conspicuity was assessed on a scale of 1 to 3: grade 1 (low conspicuity), conspicuity ratio >0.7; grade 2 (moderate conspicuity), conspicuity ratio from 0.4 to 0.7; and grade 3 (high conspicuity), conspicuity ratio <0.4.

### Statistical Analysis

All statistics analyses were performed using SPSS statistical software (version 16.0, SPSS Inc., Chicago, Illinois). P values of 0.05 were considered statistically significant. The number, size, and conspicuity of splenic siderotic nodules identified by SWI and by T1WI, T2WI and T2*WI were compared by paired *t* test. Cohen’s kappa method was used to assess inter-reader agreement.

## Results

**Figure 1:**
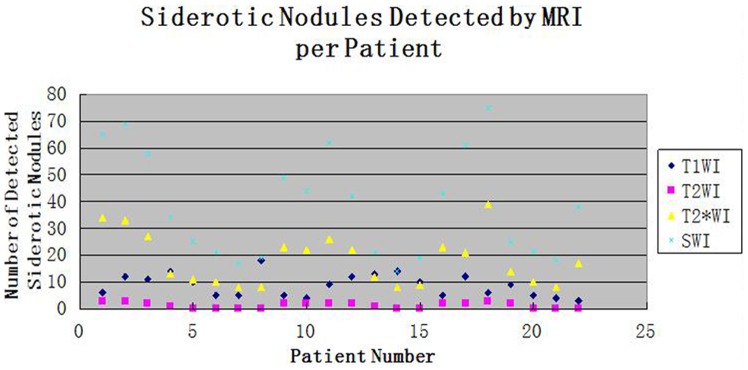
A plot of the number of siderotic nodules detected by MRI in each patient. The horizontal axis indicates the patient number, and the vertical axis indicates the number of siderotic nodules. Black rhombus, red squares, yellow triangles and blue star represent T1-weighted imaging, T2-weighted imaging, T2*-weighted imaging, and susceptibility-weighted imaging, respectively.

**Figure 2:**
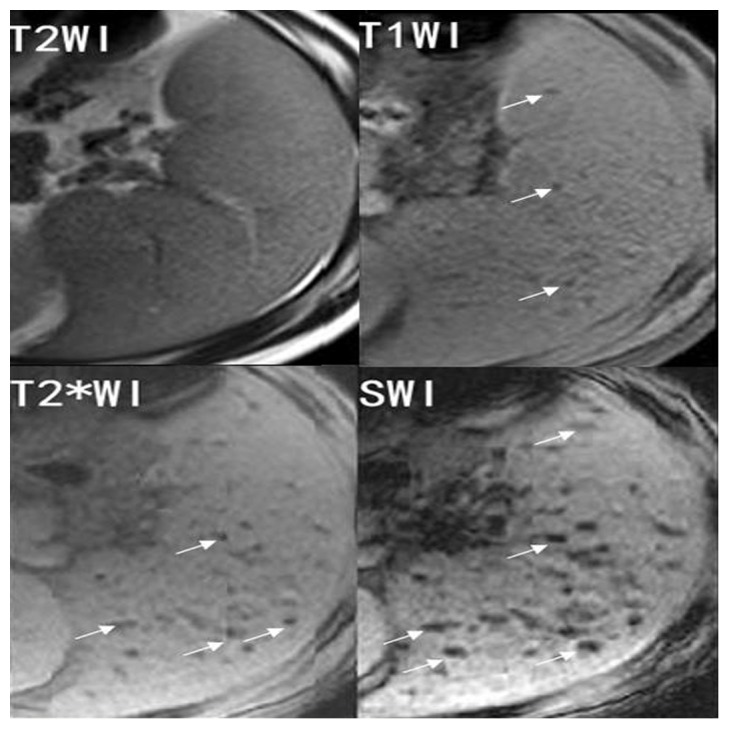
Representative T1-weighted image (T1WI), T2-weighted image (T2WI), T2*-weighted image (T2*WI) and susceptibility-weighted imaging (SWI) of a 36 year-old patient with splenic siderotic nodules. SWI shows a greater number of hypointense lesions (white arrows) with larger size and higher contrast than T1WI, T2WI or T2*WI.

**Figure 3:**
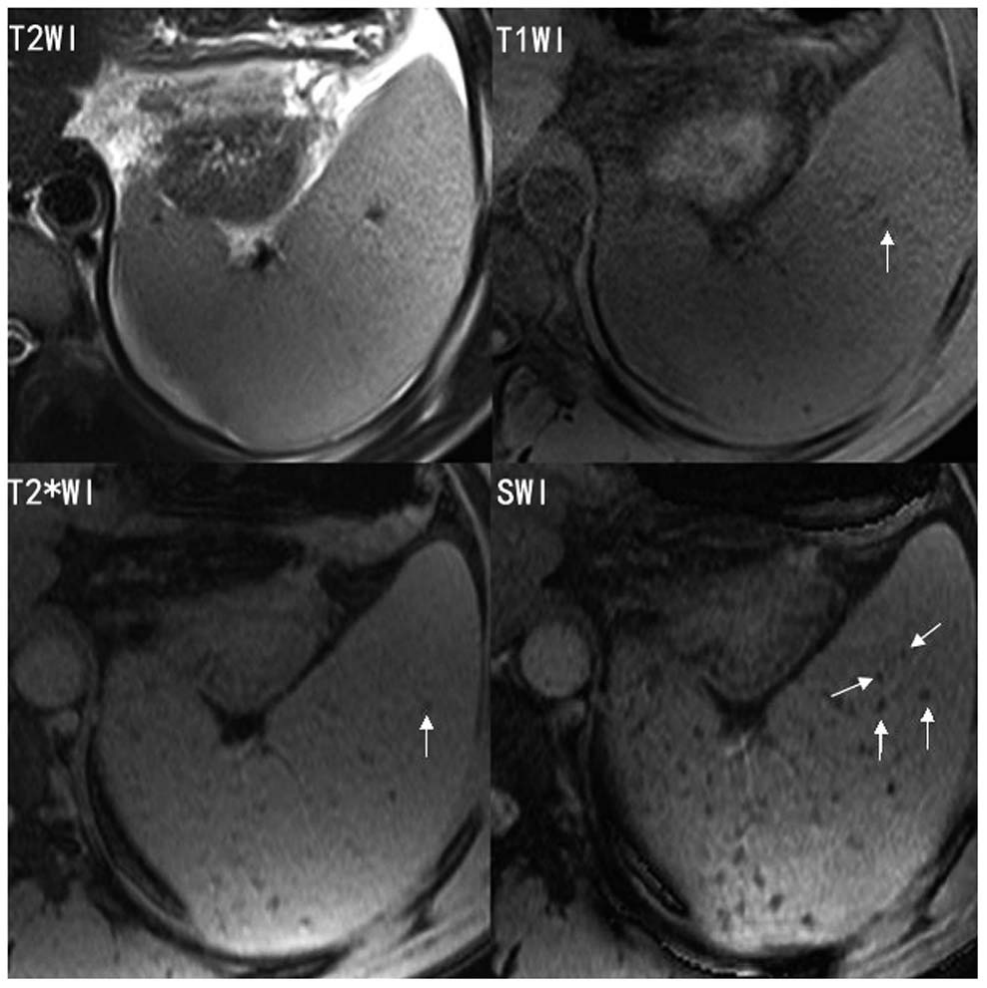
Representative T1-weighted image (T1WI), T2-weighted image (T2WI), T2*-weighted image (T2*WI) and susceptibility-weighted imaging (SWI) of a 42 year-old patient with splenic siderotic nodules. SWI shows more siderotic nodules than T1WI, T2WI or T2*WI.

The average number of splenic siderotic nodules detected by SWI (38.2±19.7) was greater than that by T1WI, T2WI or T2*WI (8.7±4.1, 1.2±0.8, or 18.1±9.5, respectively, all p<0.05). The average size of splenic siderotic nodules on SWI (2.83±1.35) was higher than that on T1WI, T2WI or T2*WI, (2.62±1.31, 1.28±0.46, or 2.21±1.06, respectively, all p<0.05) (Figures 1, 2, 3). There was no case where SWI showed a smaller siderotic nodule number or size than the other imaging techniques.

Regarding conspicuity, SWI performed significantly better than T1WI, T2WI and T2*WI (P<0.001) (Figure 2, 3). Lesions conspicuity was lowest in T2WI and greatest in SWI.

Inter-reader correlation, based on results provided by each individual reader and determined using the K coefficient, was estimated at 0.82–0.89.

## Discussion

To our knowledge, this is the first study of SWI applied to splenic siderotic nodule detection. We found that the average number and size of splenic siderotic nodules detected by SWI were greater than those detected by T1WI, T2WI or T2*WI. There was no case where SWI showed a smaller number or size than the other imaging techniques. With regards to nodule conspicuity, a statistically significant superiority of SWI over T1WI, T2WI and T2*WI was found. Splenic siderotic nodules detected on SWI had superior contrast and visibility than on any other MRI sequence. These findings suggest that SWI is powerful, efficient and the best imaging method for the identification and evaluation of spleen iron detection. In past studies, T2*WI has been used reliably to quantify myocardial and hepatic iron [10]. In this study we found SWI to be more sensitive than T2*WI. This result may be explained by the fact that phase data of SWI serves as an additional source of information about local susceptibility changes induced by iron or deoxyhemoglobin. Previous studies have shown that SWI can detect as many as 5 times more cerebral hemorrhagic lesions in patients with diffuse axonal injury than T2* weighted imaging [16].

Detection of siderotic nodules has broad medical applicability. With approximately 60,000 new cases each year, thalassemia is the most common genetic disorder worldwide. Iron overload in patients with thalassemia results from both excessive iron absorption and transfusion. Precise iron overload detection can prevent iron toxicity and avoid excessive administration of chelator [17]. Moreover, body iron burden has been shown to be the major determinant of early death in patients with thalassemia [18]–[20]. Detection of siderotic nodules could also be applied to help patients with genetic hemochromatosis, a common autosomal recessive disorder with a homozygous frequency between 0.2% and 0.45% in the white population. Determining iron overload permits evaluation of iron-induced organ damage risk for patients with hemochromatosis, who chronically absorb inappropriately large amounts of iron from the bowel [21]–[25]. A third example of patients who may be helped by the detection of siderotic nodules are patients who undergo multiple parenteral transfusions, in whom secondary iron overload may occur. The iron contained in transfused erythrocytes preferentially deposits within the spleen, liver and bone marrow, sometimes leading to organ dysfunction. Iron deposition has been associated with liver fibrosis, cirrhosis, hepatocellular carcinoma and spleen angiosarcoma [26], [27]. Improving the sensitivity of the detection of siderotic nodules could help the diagnosis and treatment of these diseases.

This study has several recognized limitations. First, this is a retrospective study with a small sample size, and only a single representative image per imaging technique was chosen for evaluation. Correspondingly, there is potential for bias. Second, not all splenic siderotic nodules shown on SWI could be verified by pathological examination. Third, because abdomen SWI is sensitive to motion artifact from respiratory movement, our use of three consecutive breath-hold acquisitions may not be feasible in all cirrhotic patients, especially those with pulmonary compromise from hepatopulmonary syndrome or ascites.

In conclusion, we demonstrated the value of SWI in improving the detection of splenic siderotic nodules. SWI may be a better detection scheme for splenic siderotic nodules than T1WI, T2WI and T2*WI. SWI should be a useful addition to imaging techniques currently in clinical practice.
